# Global burden of Alzheimer’s disease and other dementias (1990–2021): inequality, frontier, and decomposition analysis

**DOI:** 10.3389/fnagi.2025.1637029

**Published:** 2025-09-18

**Authors:** Haishou Fu, Huaqing Huang, Shuzhen Liao, Zhisen Dai

**Affiliations:** ^1^Department of Laboratory Medicine, Fuzhou University Affiliated Provincial Hospital, School of Medicine, Fuzhou University, Shengli Clinical Medical College of Fujian Medical University, Fuzhou, China; ^2^Department of Pain Medicine, Clinical Oncology School of Fujian Medical University, Fujian Cancer Hospital, Fuzhou, China; ^3^Pain Research Institute of Fujian Medical University, Fuzhou, China

**Keywords:** Alzheimer’s disease, dementia, global burden of disease, incidence, mortality, risk factors, frontier analysis

## Abstract

**Introduction:**

Alzheimer’s disease and other dementias (ADOD) represent a growing global health crisis driven by the rapid aging of the population. Using data from the 2021 Global Burden of Disease (GBD) study, we provide updated global and national estimates of the ADOD burden from 1990 to 2021, quantify key risk factors, and offer evidence to guide resource allocation and prevention strategies.

**Methods:**

We analyzed age-standardized incidence, prevalence, mortality, and disability-adjusted life years (DALYs) rates, along with the burden attributable to ADOD-related risk factors. Health disparities were assessed using the slope index of inequality and concentration index. We used frontier analysis to evaluate outcomes relative to development levels. Decomposition analysis identified the drivers of burden changes.

**Results:**

Globally, ADOD incidence, prevalence, and mortality increased significantly between 1990 and 2021 (156.54%, 160.81%, and 194.39%, respectively). High fasting blood glucose was the leading modifiable risk factor, contributing to 14.70% of global ADOD mortality, followed by elevated BMI and tobacco use. Burden trends varied across Socio-Demographic Index (SDI) regions, with high-middle SDI regions showing the highest prevalence. Gender-specific risk factor rankings showed high BMI as the second most significant factor in females, while tobacco use ranked second in males but declined over time. Decomposition analysis revealed the greatest increases in DALYs occurred in middle-SDI regions.

**Discussion:**

This study reveals a considerable potential to reduce the ADOD burden in many countries. Although some metrics have stabilized or slowed in growth, significant inequalities remain, particularly in lower-SDI nations. Tailored strategies focusing on strengthening healthcare systems, targeting high-risk populations, and improving health education are essential to narrow these gaps. Greater international collaboration and open data sharing are also key to building a life-course management model for ADOD prevention and care, ultimately improving global health.

## 1 Introduction

Alzheimer’s disease and related dementias (ADOD) represent a growing global health challenge, characterized by progressive neurodegeneration that initially manifests as mild cognitive impairments, particularly affecting memory, language, and executive functions (Matthews et al., 2019; Mobaderi et al., 2024). Early manifestations, though often subtle, may include forgetfulness, difficulty in word finding, and reduced problem-solving skills, which progressively escalate over time (Li et al., 2022). These changes eventually lead to substantial cognitive and functional decline, disrupting independent living and increasing reliance on caregivers. As the disease progresses, it can impair fundamental physical abilities, including walking and swallowing, thereby necessitating long-term institutional or home-based care (Reiman et al., 2012; Villemagne et al., 2013). The societal and economic consequences are profound, with dementia care costs projected to exceed US$2 trillion globally by 2050 (Lastuka et al., 2024). With no known cure, the rising prevalence of ADOD, coupled with an aging global population, poses urgent challenges for public health systems, caregivers, and policy-makers worldwide.

Population aging is a prominent and ongoing demographic shift that poses substantial challenges to global healthcare infrastructures (Jack et al., 2009). In 2019, the world was home to 703 million individuals aged 65 years or older. This number is expected to more than double by 2050, with one-sixth of the global population reaching this age group (Jack et al., 2009). This demographic transformation will not only increase healthcare demand but also magnify inequalities in dementia care, especially in low- and middle-income countries where healthcare resources are already limited.

The global prevalence of ADOD has increased dramatically over the past three decades. In 2019, an estimated 51.62 million individuals were living with dementia, marking a staggering 160.84% increase since 1990 (Li et al., 2022). Without effective preventive strategies, the number is expected to rise to 152 million by 2050 (GBD 2019 Dementia Forecasting Collaborators, 2022). As life expectancy increases, the proportion of the population aged 60 years and older is projected to nearly double, from 12% in 2015 to 22% by 2050 (Zhang et al., 2023). Given that age remains the strongest risk factor for dementia onset, this aging trend will directly accelerate the ADOD burden. With AD remaining ultimately fatal and an average survival of 4 to 8 years post-diagnosis (Fu et al., 2024), the growing prevalence of ADOD will continue to strain healthcare systems, increase caregiver burden, and widen disparities in diagnosis and treatment access across regions.

Despite substantial research progress, significant knowledge gaps remain regarding the drivers of geographical inequalities in ADOD burden, the role of socioeconomic development, and the interaction between modifiable risk factors (Livingston et al., 2020). This study addresses these gaps by exploring the evolving trends in ADOD from 1990 to 2021, focusing on global, regional, and national disparities by gender, age, and geography. By leveraging the Global Burden of Disease (GBD) 2021 data, we assess key risk factors contributing to the growing ADOD burden across various SDI regions, with the aim of providing evidence to guide targeted interventions, resource allocation, and policy development for dementia prevention and care.

## 2 Method

### 2.1 Data source

The GBD study is a comprehensive effort to quantify the impact of 371 diseases, injuries, and 88 risk factors on global health, providing an essential resource for understanding trends in incidence, prevalence, mortality, and disability-adjusted life years (DALYs) related to ADOD (GBD 2021 Causes of Death Collaborators, 2024). The work has been reported in line with the STROCSS criteria (Rashid et al., 2024).

ADOD data for this study were obtained from the most recent Global Health Data Exchange (GHDx) Query Tool,^1^ accessed on January 25, 2025, using the GBD 2021 release. We explicitly documented the search strategy, including disease category (“Alzheimer’s disease and other dementias”), metrics (incidence, prevalence, mortality, DALYs), and population filters, to ensure full reproducibility.

This cross-sectional study investigates ADOD, encompassing conditions such as vascular dementia, HIV-associated dementia, and others. The analysis utilized data from 1990 to 2021, covering all age groups. However, due to the absence of data on the ADOD burden for individuals under 40 years old in GBD 2021, the study primarily focused on individuals aged 40 years and older. The analysis was conducted at global, regional, and country levels, with particular attention to differences across sexes and age ranges. The 21 regions in the GBD 2021 classification encompass diverse areas, including Andean Latin America, Australasia, the Caribbean, Central Asia, Central and Eastern Europe, and Latin America. It also covers regions from Sub-Saharan Africa, such as Central, Eastern, and Southern Sub-Saharan Africa, alongside high-income regions like Asia Pacific and North America. Additionally, regions from the Middle East, Oceania, South Asia, Southeast Asia, and various parts of Latin America, such as Southern Latin America and Tropical Latin America, were included. Furthermore, Western Europe and Western Sub-Saharan Africa are also represented. This classification system has been consistently applied in previous GBD iterations and remains effective in comparing health metrics across distinct areas (GBD 2021 Nervous System Disorders Collaborators, 2024). Using their analytical modeling approach, the GBD generated 1,000 estimates for each value to calculate the 95% uncertainty intervals (UI). The lower and upper bounds of these intervals were then established by the 25th and 975th ranked values from this ordered set of estimates. Key trends in incidence, prevalence, mortality, and Disability-Adjusted Life Years (DALYs)—a metric that combines years of life lost due to premature mortality and years lived with disability—were analyzed over the study period.

The sociodemographic index (SDI) is a composite metric used to evaluate a location’s socioeconomic status. It integrates data on three key factors: per capita income, the average educational attainment of individuals aged 15 and older, and the total fertility rate for those younger than 25 (GBD 2021 Nervous System Disorders Collaborators, 2024). In the GBD 2021 study, 204 countries and territories were categorized into five groups—low (< 0.46), low-middle (0.46–0.60), middle (0.61–0.69), high-middle (0.70–0.81), and high (> 0.81)—based on their respective Sociodemographic Index (SDI) values.

The standardized population used in this study was based on the world standard population compiled by GBD 2021, using a direct standardization method to age-standardize morality from high BMI leading to ADOD. High BMI in adults (age 18+ years) was defined as BMI ≥ 25 kg/m^2^ (Du et al., 2022).

### 2.2 Incidence, prevalence, mortality, DALYs and risk estimates

In this study, we gathered data on the number of cases, incidence, prevalence, mortality, and DALYs of ADOD, along with their corresponding rates at global, regional, and national levels. We calculated the mean estimated annual percentage changes (EAPCs) using a log-linear regression model, which is widely applied in GBD trend analyses due to its ability to estimate the average annual rate of change in age-standardized rates under the assumption of exponential growth or decline. Age-standardized rates (ASRs) were used to control for age-related confounding and ensure consistency in rate comparisons. The GBD 2021 companion paper thoroughly describes the data inputs, processing methods, synthesis, and modeling approach used to estimate disease burden (GBD 2021 Causes of Death Collaborators, 2024).

The GBD 2021 analysis of risk factors adhered to the comparative risk assessment framework, which involved identifying risk-outcome pairs, estimating relative risks, assessing exposure levels and distributions, defining counterfactual exposure levels, calculating population attributable fractions, and determining the attributable burden of diseases. It also included estimating the mediating effects of various risk factors through other risk factors. We referenced WHO guidelines for risk factor definitions,^2^ and clarified that high fasting blood glucose, high body mass index (BMI), and tobacco use were the primary risk factors identified for dementia in previous literature (Li et al., 2022).

### 2.3 Cross-country inequality analysis

This study assessed absolute and relative inequalities in ADOD burden using the slope index of inequality (SII) and the concentration index, as defined by the WHO (Zhang et al., 2023). The SII was calculated by regressing the DALYs rate on the SDI, considering the midpoint of the cumulative population distribution ranked by SDI. We explicitly chose the SII and concentration index because they allow for quantifying inequality across the entire socioeconomic distribution, rather than only comparing extremes, which is crucial for assessing health disparities (Erreygers and Van Ourti, 2011). A robust regression model (rlm) was employed to minimize bias from data heterogeneity and outliers, offering a more accurate representation of health inequalities compared to ordinary linear regression. The concentration index was derived by integrating the area under the Lorenz curve, matching the cumulative proportion of DALYs with the cumulative population distribution by SDI. Data from 204 countries and territories between 1990 and 2021 were analyzed to examine changes in ADOD-related inequalities.

### 2.4 Frontier analysis and decomposition analysis

This study used frontier analysis to evaluate the relationship between the burden of ADOD and sociodemographic development levels, constructing an age-standardized DALYs rate (ASDR)-based frontier model with SDI. We selected this method because frontier analysis can identify the theoretical minimum burden achievable at each development level, highlighting efficiency gaps, as demonstrated in previous GBD studies (Murray et al., 2018). Frontier analysis, unlike traditional regression models, captures the nonlinear relationship between SDI and disease burden, identifying the theoretical minimum ASDR achievable by each country or territory based on its development level. The analysis quantified the gap between current and potential burden, highlighting areas for improvement. Locally weighted regression (LOESS) and local polynomial regression were applied with multiple smoothing spans to create smooth frontier lines, supported by 1,000 bootstrap samples for robustness. Improvement potential was assessed by measuring the distance between each country’s 2021 ASDR and the frontier line (Xie et al., 2018).

Decomposition analysis was performed using the Das Gupta method to break down changes in ADOD burden from 1990 to 2021 into contributions from aging, population growth, and epidemiological changes. This approach provided detailed insights into how these factors independently shaped trends over time, enabling a clearer understanding of the drivers of changes in the global ADOD burden (He et al., 2024; United States Census Bureau, 1993).

### 2.5 Ethics approval and consent to participate

The university’s ethical board granted a waiver of informed consent, as the study involved only data analysis and did not include identifiable personal information.

### 2.6 Statistical analysis

We described the numbers and trends of incident cases, prevalent cases, mortality, and DALYs for ADOD, along with their corresponding ASRs and associated 95% uncertainty intervals (UIs), stratified by sex, age, year, SDI subregion, GBD regions, and 204 countries and territories. The time trends of ASRs in a specific time period are represented by the EAPC. In short, we used the y=a+βx+∈ regression model for these calculations, where y is ln (ASR), x is the time variable, and ∈ is the error term. The natural logarithm of the ASR was assumed to be linear with time; therefore, EAPC=100×[exp(β)−1] (GBD 2019 Dementia Forecasting Collaborators, 2022). In this study, figures were created using R software (version 4.2.3) and JD_GBDR (V2.25, Jingding Medical Technology Co., Ltd.).

## 3 Result

### 3.1 The global ADOD-related disease burden

**Figure 1:**
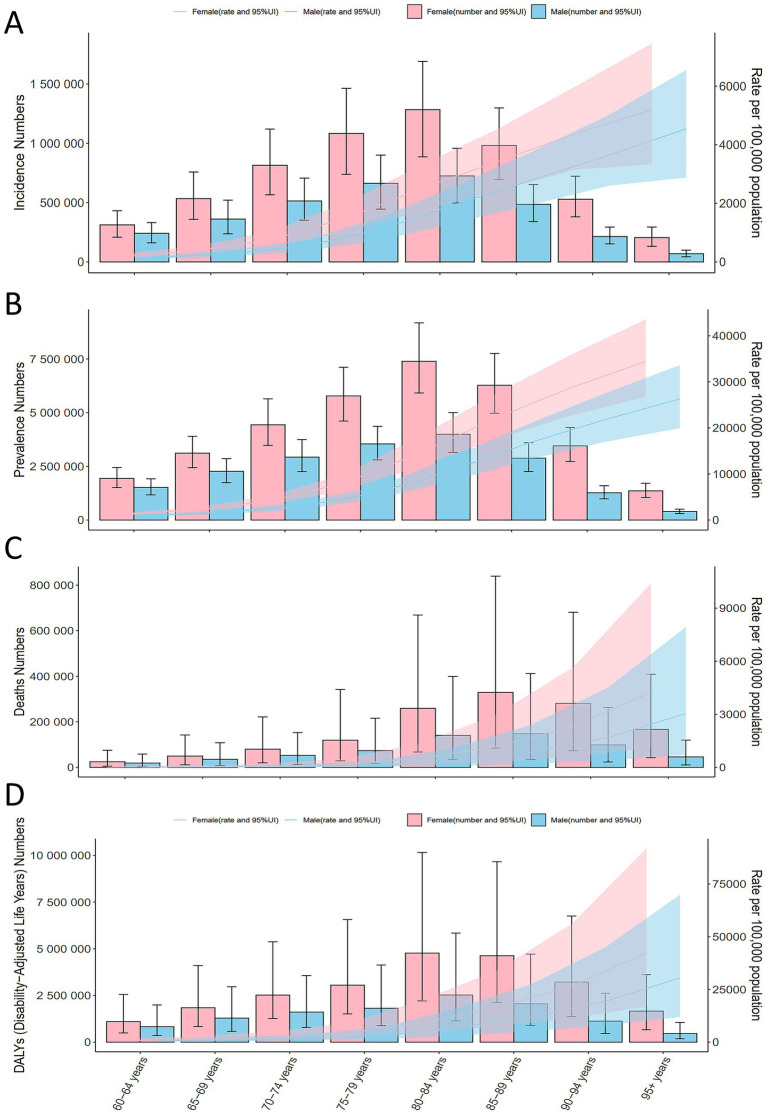
Global ADOD incidence, prevalence, mortality, and DALYs by age in 2021. **(A)** Global ADOD incidence by age in 2021. **(B)** Global ADOD prevalence by age in 2021. **(C)** Global ADOD mortality by age in 2021. **(D)** Global ADOD DALYs by age in 2021. ADOD, Alzheimer’s disease and other dementias; DALYs, Disability-Adjusted Life-Years.

From 1990 to 2021, the global number of ADOD patients increased by 155.27% to 56.86 million, with consistently higher incidence in females across all age groups (Table 1). The highest incidence was observed in females aged ≥95 years (5196.53 per 100,000; 95% UI: 3334.49–7451.56) (Figure 1, Supplementary Table S1). Global mortality nearly tripled over the study period, with both sexes showing increased age-standardized mortality rates (ASMR). The ASPR in 2021 was 694.01 per 100,000 (95% UI: 602.88–794.08), with an EAPC of 0.00 (95% CI: −0.02 to 0.03), indicating a stable trend (Supplementary Table S2).

**Table 1:** Incidence and ASIR of ADOD in 1990 vs. 2021 and EAPC analysis.

Characteristics	1990		2021		1990–2021
	Incidence cases (95%UI)	ASIR (per 100,000) (95%UI)	Incidence cases (95%UI)	ASIR (per 100,000) (95%UI)	EAPC of ASIR (95% CI)
Global	3834525.86 (3367544.12–4358427.97)	116.97 (102.77–132.32)	9837055.84 (8620519.20–11163699.62)	119.76 (104.96–135.89)	-0.02 (−0.04–0.00)
Gender					
Females	2482206.92 (2183968.02–2820920.01)	127.82 (112.82–144.18)	6191564.17 (5432752.48–7009225.95)	132.29 (116.30–149.80)	0.00 (−0.03–0.03)
Males	1352318.94 (1177679.88–1551793.07)	100.69 (88.05–114.43)	3645491.67 (3144737.84–4183541.38)	103.40 (89.45–118.45)	0.02 (−0.00–0.03)
Sociodemographic index					
High SDI	1435427.42 (1264748.77–1624789.09)	127.18 (112.55–142.92)	2952146.94 (2586722.06–3344548.06)	122.61 (107.45–138.44)	−0.11 (−0.12--0.10)
High-middle SDI	990611.65 (862203.22–1138429.23)	118.38 (103.82–134.11)	2582346.16 (2252028.81–2941774.29)	132.40 (115.43–150.85)	0.22 (0.18–0.26)
Middle SDI	844508.80 (735191.04–963600.79)	113.27 (99.18–128.90)	2902079.92 (2542319.83–3314767.30)	123.79 (108.25–141.26)	0.09 (0.05–0.14)
Low-middle SDI	415381.55 (363221.76–471142.90)	95.55 (83.27–108.90)	1063277.08 (929611.91–1207789.69)	92.61 (80.79–105.71)	−0.17 (−0.18--0.15)
Low SDI	144165.00 (125717.92–163571.61)	95.08 (82.74–108.32)	328702.71 (287112.20–372980.85)	90.89 (79.00–103.12)	−0.19 (−0.20--0.17)
21 GBD regions					
Andean Latin America	14094.02 (12247.55–15984.38)	80.60 (69.91–92.12)	44485.77 (38612.47–50841.31)	79.55 (68.95–91.05)	−0.06 (−0.07--0.05)
Australasia	28408.43 (24870.92–32324.86)	123.79 (108.48–139.61)	63288.53 (55457.11–71346.59)	105.44 (92.56–118.63)	−0.56 (−0.60--0.52)
Caribbean	22906.71 (19921.60–26131.69)	97.60 (85.15–110.90)	52399.68 (45969.58–59446.22)	95.60 (83.53–108.64)	−0.16 (−0.19--0.13)
Central Asia	44784.25 (39050.74–51312.94)	111.72 (97.73–127.69)	69754.83 (61205.22–79094.11)	109.82 (95.87–125.30)	−0.06 (−0.07--0.04)
Central Europe	152640.45 (131915.20–177170.11)	115.01 (100.69–131.62)	269693.15 (233101.96–309820.37)	112.42 (97.97–128.46)	−0.08 (−0.09--0.07)
Central Latin America	74979.86 (65388.91–85453.92)	111.25 (96.71–126.56)	248660.86 (218264.63–282093.90)	106.12 (92.53–120.99)	−0.11 (−0.12--0.09)
Central Sub-Saharan Africa	17483.25 (15147.84–19938.27)	126.90 (111.06–144.40)	44061.65 (38634.35–49576.49)	126.14 (111.31–143.21)	−0.01 (−0.04–0.01)
East Asia	725924.64 (621289.73–834629.72)	120.29 (104.75–137.02)	2988724.23 (2569166.05–3434391.97)	149.61 (129.58–171.14)	0.40 (0.33–0.48)
Eastern Europe	291032.54 (251713.00–335607.18)	117.31 (102.49–133.78)	416088.85 (361581.06–476873.86)	115.66 (100.94–131.87)	−0.08 (−0.11--0.04)
Eastern Sub-Saharan Africa	53366.47 (46292.32–60685.77)	107.12 (93.44–121.63)	121209.89 (106416.00–137289.38)	102.41 (89.70–116.09)	−0.13 (−0.14--0.12)
High-income Asia Pacific	212521.89 (185436.08–242783.57)	116.94 (102.53–133.08)	701760.50 (614636.07–802668.29)	118.62 (103.43–135.00)	0.19 (0.14–0.24)
High-income North America	520200.45 (454539.55–593017.13)	139.57 (122.14–157.89)	928259.16 (812567.48–1051337.97)	131.39 (114.67–149.04)	−0.22 (−0.24--0.20)
North Africa and Middle East	170210.67 (149710.12–192327.57)	138.06 (121.37–156.98)	468045.33 (411549.98–530963.36)	132.19 (115.75–150.35)	−0.14 (−0.15--0.13)
Oceania	2057.89 (1762.17–2366.13)	117.17 (102.35–133.78)	5421.89 (4714.66–6169.14)	112.05 (96.98–128.81)	−0.18 (−0.21--0.16)
South Asia	315180.73 (273889.36–359432.93)	80.57 (69.50–92.11)	924833.04 (799983.38–1056260.56)	79.00 (68.26–90.52)	−0.18 (−0.22−−0.14)
Southeast Asia	216530.18 (189078.65–245887.29)	114.85 (100.81–130.85)	578242.10 (505926.68–658512.73)	110.07 (96.11–125.72)	−0.14 (−0.15--0.12)
Southern Latin America	46975.07 (40636.01–53790.22)	111.77 (97.43–127.36)	98313.16 (85032.14–112618.00)	107.09 (92.90–122.43)	−0.14 (−0.15--0.13)
Southern Sub-Saharan Africa	24227.19 (21094.07–27411.04)	112.76 (98.34–128.64)	47444.30 (41315.69–54029.13)	107.13 (93.16–122.19)	-0.14 (−0.15--0.12)
Tropical Latin America	92945.51 (81381.28–105405.43)	129.14 (113.10–146.66)	311555.99 (274795.89–352659.10)	126.83 (111.77–144.42)	−0.11 (−0.15--0.08)
Western Europe	758286.54 (671203.93–848021.60)	122.45 (108.70–136.67)	1352813.32 (1182307.55–1543865.98)	118.56 (103.36–134.23)	−0.14 (−0.18--0.11)
Western Sub-Saharan Africa	49769.11 (43260.59–56437.70)	78.42 (67.93–89.46)	101999.61 (88928.41–115137.19)	73.18 (63.36–83.46)	−0.23 (−0.24--0.21)

ADOD, Alzheimer’s disease and other dementias; EAPC, estimated annual percentage change.

Between 1990 and 2021, global mortality due to ADOD nearly tripled, increasing from 0.66 million (95% UI: 0.16–0.17) to 1.95 million (95% UI: 0.51–4.98) (Supplementary Table S3). ASMR also rose, with an EAPC of 0.02 (95% CI: 0.00–0.03). Both sexes experienced rising ASMRs, with EAPCs of 0.06 (95% CI: 0.04–0.07) for males and 0.04 (95% CI: 0.02–0.06) for females (Supplementary Table S3, Supplementary Figure 1).

Although the age-standardized DALY rate (ASDR) showed a slight decline (EAPC: −0.02; 95% CI: −0.03 to −0.00) (Supplementary Table S4), mortality rates and DALYs increased with age. The fastest growth in deaths occurred in the >95 age group, while the 80–84 age group experienced the most rapid increase in DALYs (Figure 1).

### 3.2 SDI and the ADOD-related disease burden

**Figure 2:**
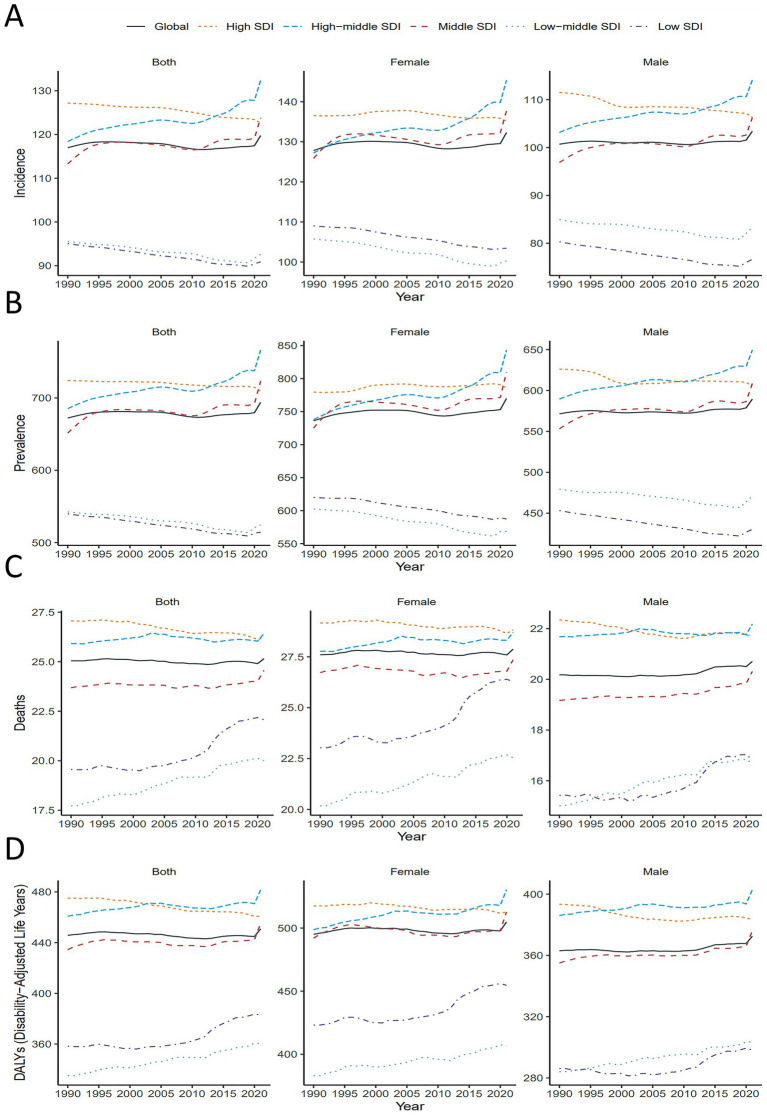
Global and 5 SDI regions ASIR, ASPR, ASMR and ASDR of ADOD by sex, 1990–2021. **(A)** Global and 5 SDI regions ASIR of ADOD by sex, 1990–2021. **(B)** Global and 5 SDI regions ASPR of ADOD by sex, 1990–2021. **(C)** Global and 5 SDI regions ASMR of ADOD by sex, 1990–2021. **(D)** Global and 5 SDI regions ASDR of ADOD by sex, 1990–2021. ADOD, Alzheimer’s disease and other dementias; ASDR, age-standardized disability-adjusted life years rate; ASIR, age-standardized incidence rate; ASMR, age-standardized mortality rate; ASPR, age-standardized prevalence rate; DALY, disability-adjusted life-years.

204 countries were grouped into five SDI categories: high (>0.81), medium-high (0.70–0.81), medium (0.61–0.70), medium-low (0.46–0.61), and low (<0.46). ASIR and ASPR showed similar patterns, with ASIR decreasing in high-SDI regions but increasing in high-middle-SDI regions (Figure 2). In 2021, ASIR was highest in high-middle-SDI regions (132.40; 95% UI, 115.43–150.85), which also had the largest ASIR increase (EAPC, 0.22; 95% CI, 0.18–0.26). In contrast, ASIR decreased in low-SDI (EAPC, −0.19; 95% CI, −0.20 to −0.17) and low-middle-SDI (EAPC, −0.17; 95% CI, −0.18 to −0.15) regions (Table 1). ASPR was also highest in high-middle-SDI regions (766.20; 95% UI, 659.80–879.64), with the fastest increase observed in the same category (EAPC, 0.21; 95% CI, 0.17–0.25). Women consistently showed higher ASPR and faster increases compared to men (EAPC, 0.04; 95% CI, 0.01–0.06).

In 2021, ASMR was highest in high-middle-SDI regions (26.42; 95% UI, 7.01–68.31), with the largest ASMR increase in low-middle-SDI regions (EAPC, 0.43; 95% CI, 0.42–0.44). Between 1990 and 2021, ASDR increased in all SDI regions except high-SDI, with the most significant rise in low-SDI regions (EAPC, 0.24; 95% CI, 0.18–0.29). In 2021, the highest ASDR was in high-middle-SDI regions (481.70; 95% UI, 157.71–754.37). Across all SDI regions, women exhibited consistently higher ASDR than men (Figure 2).

### 3.3 Global ADOD-related disease burden among 204 countries

**Figure 3:**
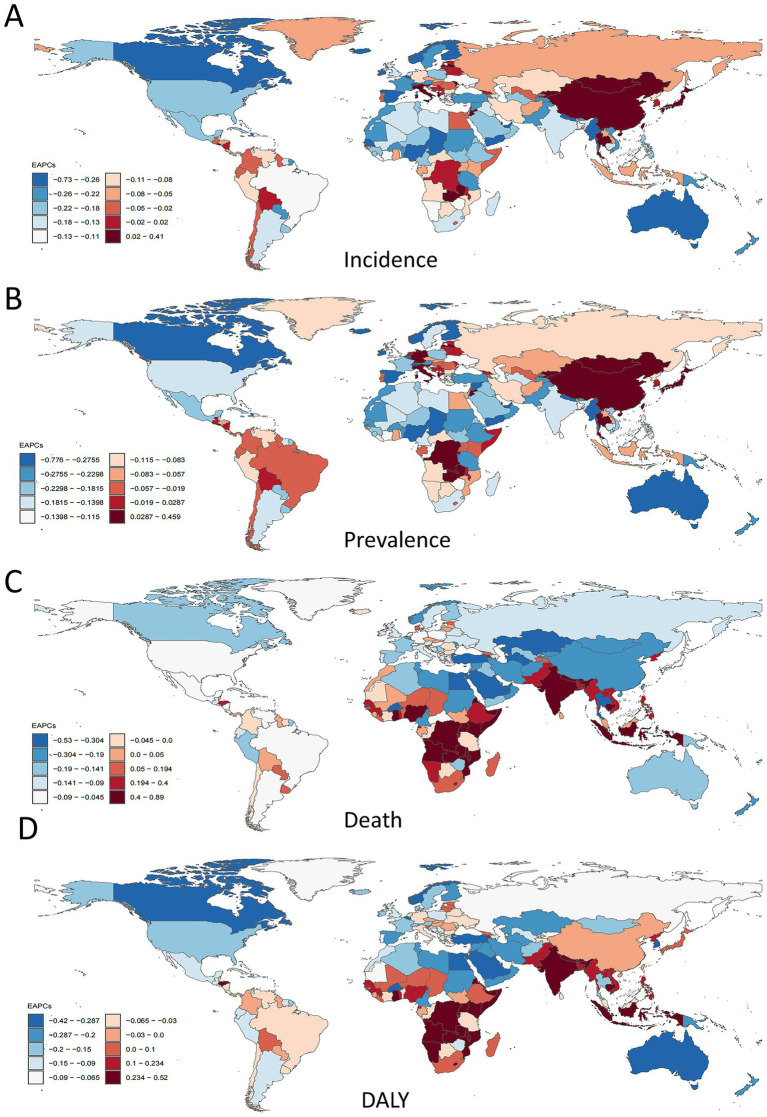
EAPC in ADOD incidence, prevalence, mortality, and DALYs across 204 countries. **(A)** EAPC in ADOD incidence, 1990–2021. **(B)** EAPC in ADOD prevalence, 1990–2021. **(C)** EAPC in ADOD mortality, 1990–2021. **(D)** EAPC in ADOD DALYs, 1990–2021. ADOD, Alzheimer’s disease and other dementias; EAPC, estimated annual percentage change; DALYs, Disability-Adjusted Life-Years.

In 2021, China, Germany, and Lebanon had the highest ASIR and ASPR of ADOD among 204 countries. China and Italy showed the fastest ASIR increases (EAPC: 0.41, 95% CI 0.33–0.49; 0.28, 95% CI 0.09–0.47), while Denmark, Norway, and Switzerland experienced the largest declines (EAPC: −0.73, −0.64, −0.34, respectively) (Supplementary Table S5, Figure 3).

Taiwan, China, and Japan exhibited the fastest ASPR increases (EAPC: 0.46, 0.44, 0.30, respectively), whereas Denmark, Norway, and Australia had the steepest declines (EAPC: −0.78, −0.67, −0.60, respectively) (Supplementary Table S6, Figure 3).

For mortality, Libya and Côte d’Ivoire recorded the highest ASMR in 2021, while Tokelau, Niue, and Nauru had the lowest. India, Bhutan, and Eritrea showed the largest ASMR increases (EAPC: 0.89, 0.85, 0.73, respectively), whereas Guam, Korea, and Bahrain experienced the greatest decreases (EAPC: −0.70, −0.53, −0.49, respectively) (Supplementary Tables S7, S8, Figure 3).

When analyzing DALYs, the highest rates in 2021 were seen in Afghanistan, Gabon, and Congo (Supplementary Table S8, Figure 3). Since 1990, the ASR of DALYs has significantly increased in India (EAPC, 0.52; 95% CI, 0.48 to 0.56) and Uganda (EAPC, 0.49; 95% CI, 0.45 to 0.52). Conversely, countries such as Guam, the United Arab Emirates, and Turkey exhibited clear downward trends (Figure 3).

### 3.4 Health inequality analysis

**Figure 4:**
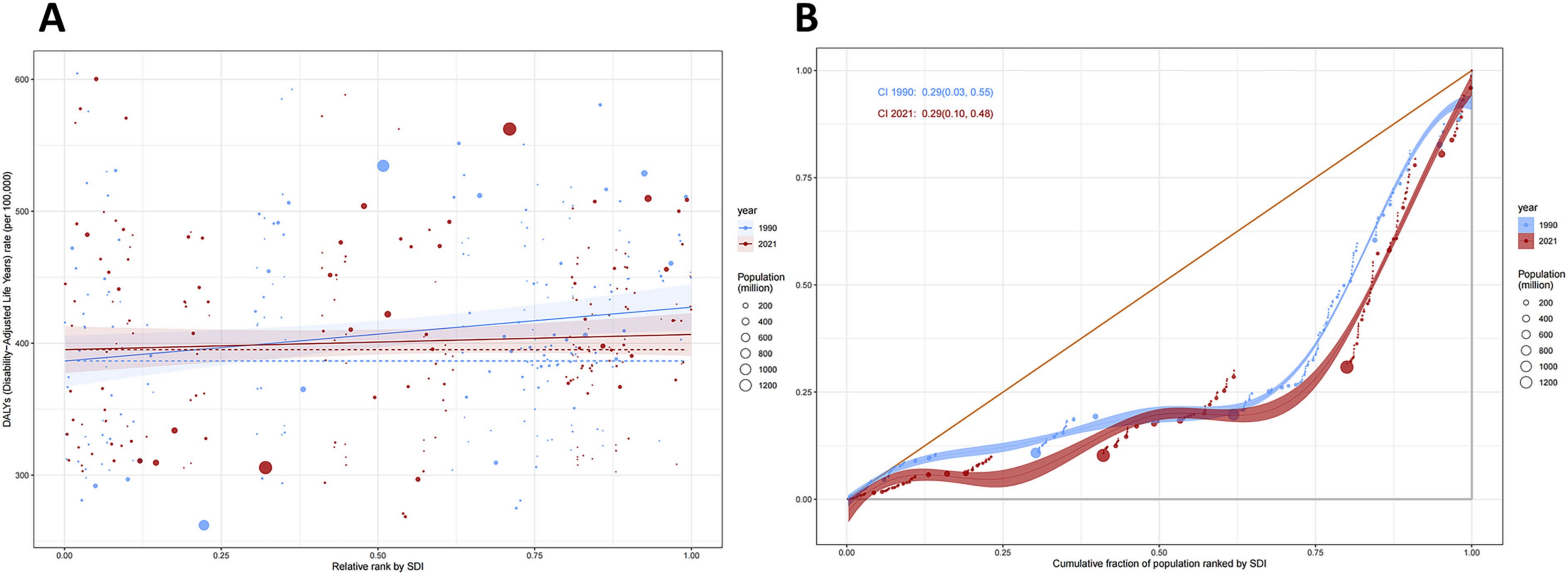
Health inequality regression curves and concentration curves for the DALYs of ADOD worldwide, 1990 and 2021. **(A)** Illustrate the slope index of inequality, depicting the relationship between SDI and age-standardized DALYs rates for each condition, with points representing individual countries and territories sized by population. **(B)** Present the concentration index, which quantifies relative inequalities by integrating the area under the Lorenz curve, aligning DALYs distribution with population distribution by SDI. Blue represents data from 1990, and red represents data from 2021. DALYs, disability-adjusted life-years; ADOD, Alzheimer’s disease and other dementias; SDI, sociodemographic index.

In terms of the burden of ADOD, we observed significant absolute and relative inequalities associated with SDI, with higher SDI countries and territories disproportionately bearing a greater burden (Figure 4). As indicated by the slope index of inequality (SII), the gap in DALY rates between the highest and lowest SDI countries and territories decreased from 40.81 (95% CI: 9.63 to 72.00) in 1990 to 11.52 (95% CI: −17.07 to 40.11) in 2021. The concentration index remained nearly unchanged, with values of 0.29 (95% CI: 0.03 to 0.55) in 1990 and 0.29 (95% CI: 0.01 to 0.48) in 2021. These findings suggest that, while absolute inequalities in the burden of ADOD have decreased from 1990 to 2021, relative inequalities have remained stable, with higher SDI countries continuing to bear a disproportionate share of the burden.

### 3.5 Frontier analysis

**Figure 5:**
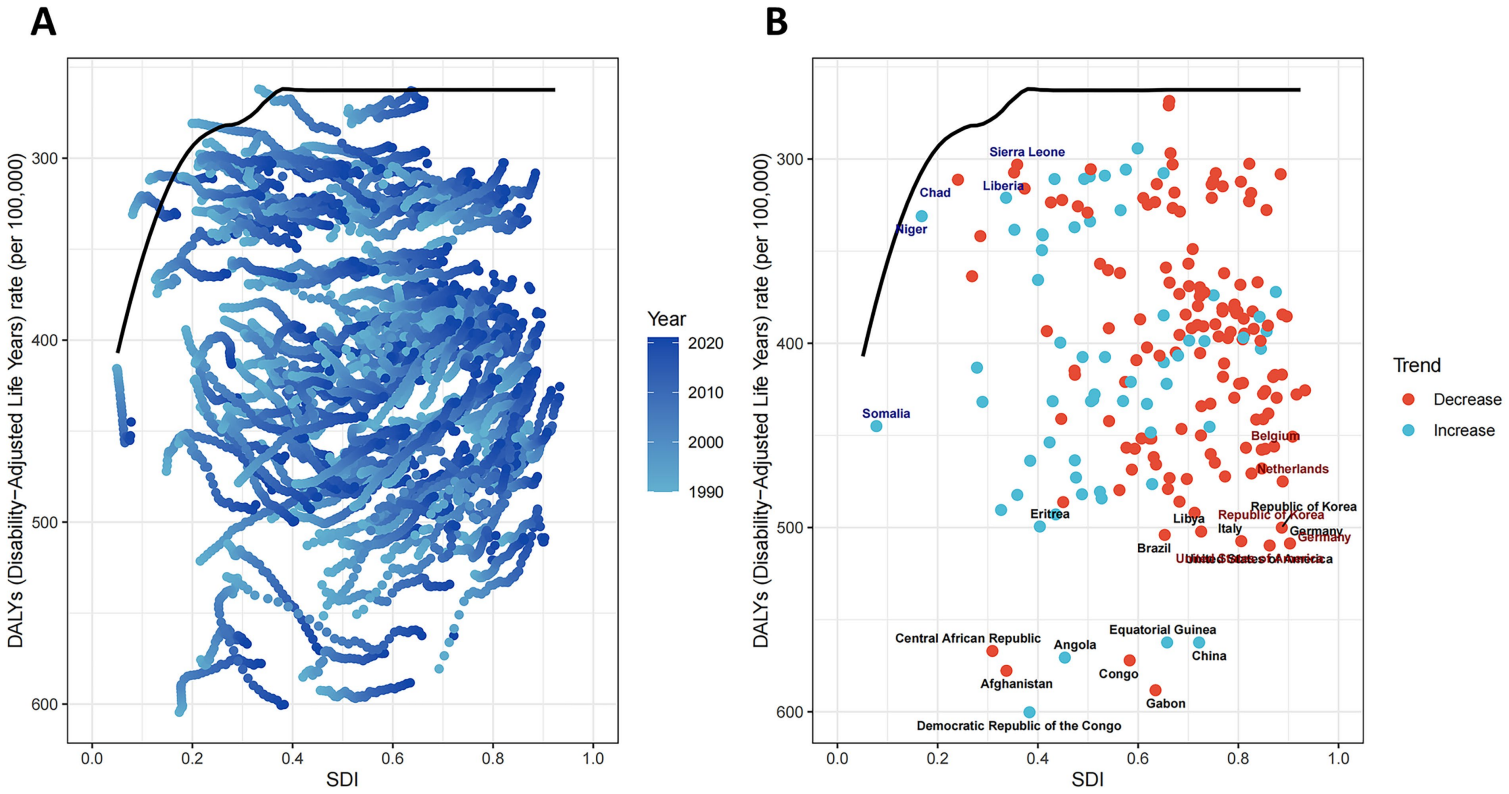
Frontier analysis exploring the relationship between SDI and ASDR for ADOD. In **(A)**, the color change from light blue (1990) to dark blue (2021) represents the change in years. In **(B)**, each point represents a specific country or territory in 2021, the frontier line is shown in black, and the top 15 countries and territories with the largest differences from the frontier are marked. The direction of ASDR change from 1990 to 2021 is indicated by the color of the dots, with orange dots representing decreases and green dots representing increases. ADOD, Alzheimer’s disease and other dementias.

Using data from 1990 to 2021 and based on ASDR and SDI, frontier analysis was conducted to assess the potential for improvement in ASDR for ADOD, considering national and regional development levels (Supplementary Table S9, Figure 5). The 15 countries with the largest actual differences in potential improvement (effective difference range: 436.83–530.87) include Eritrea, Libya, Brazil, United States of America, Central African Republic, Angola, Afghanistan, Congo, Equatorial Guinea, China, Gabon, Republic of Korea, Italy, Germany, and the Democratic Republic of the Congo. Among the low-SDI countries, Sierra Leone, Chad, Niger, Liberia, and Somalia demonstrate the largest improvement potential, while high-SDI countries such as Belgium, Netherlands, Germany, Republic of Korea and the United States of America show relatively higher improvement potential based on their development levels. Frontier analysis highlights the potential for reducing ADOD burden across countries and territories.

### 3.6 Decomposition analysis

**Figure 6:**
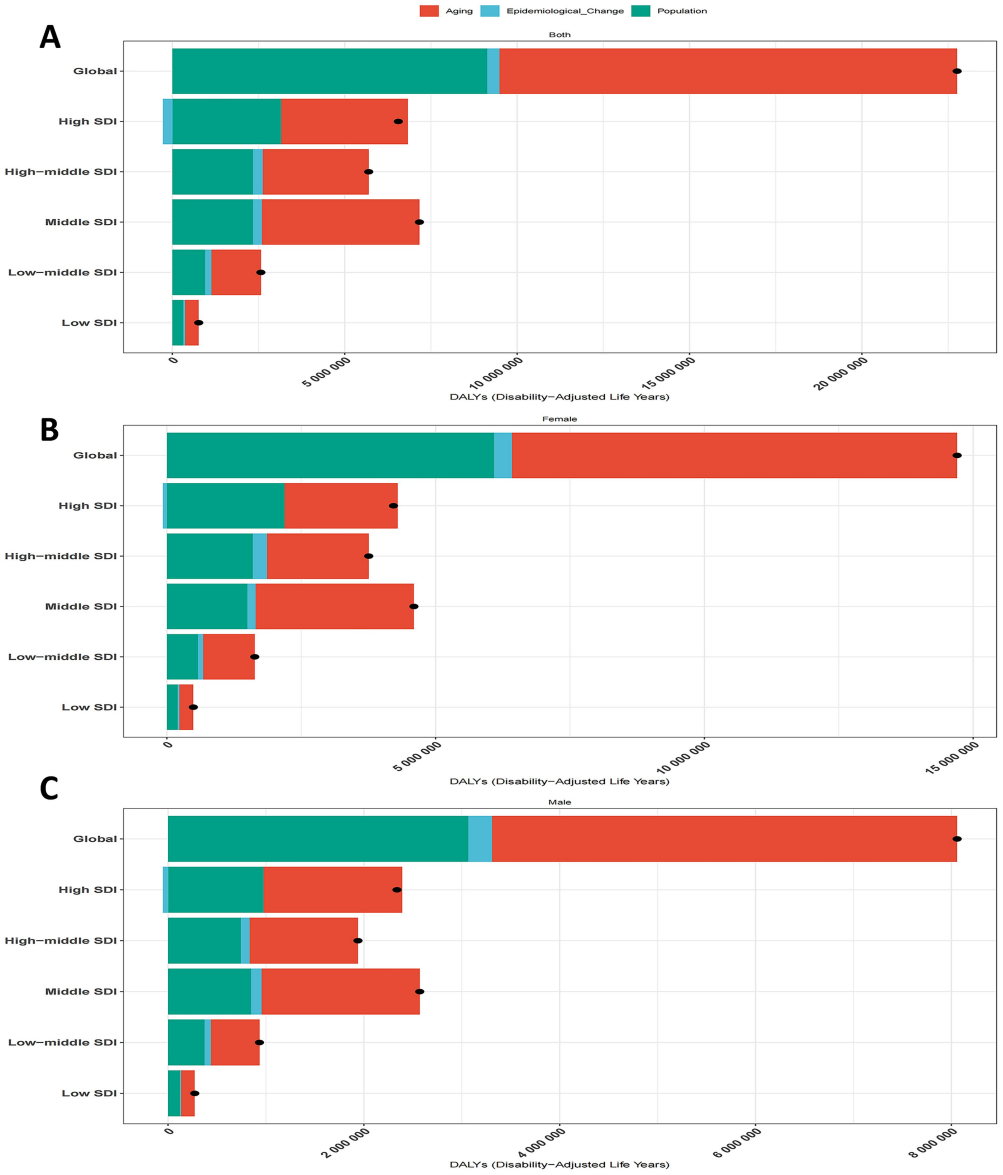
Population-level determinant changes in aging, population growth, and epidemiological changes for ADOD DALYs globally and in various SDI regions by sex from 1990 to 2021. Black dots represent the total change contributed by all three components. A positive value for each component indicates a corresponding positive contribution in DALYs, and a negative value indicates a corresponding negative contribution in DALYs. **(A)** Total change for all three components, 1990–2021. **(B)** Change in females, 1990–2021. **(C)** Change in males, 1990–2021. ADOD, Alzheimer’s disease and other dementias; SDI, sociodemographic index; DALYs, disability-adjusted life-years.

By conducting decomposition analysis on the DALYs of ADOD, this study evaluated the influence of factors such as aging, population growth, and epidemiological changes on the epidemiology of ADOD from 1990 to 2021 (Supplementary Table S10, Figure 6). Overall, DALYs for ADOD exhibited an upward trend globally and across all SDI regions, with the most significant increase observed in middle-SDI regions. On a global scale, aging and population growth contributed 58.13 and 40.14%, respectively, to the increase in disease burden. The impact of epidemiological changes varied across SDI regions, with a contribution of 5.81% in low-SDI regions, and 6.93, 3.46, 4.91, and −4.16% in low-middle, middle, middle-high, and high-SDI regions, respectively. For both males and females, aging and population growth exhibited similar trends. Epidemiological changes played a key role in the reduction of the global disease burden, especially in high-SDI regions, where they contributed −1.75% for males and −2.24% for females (Supplementary Table S10, Figure 6). The decomposition analysis indicates that aging is the primary driver behind the increase in the global ADOD burden, particularly in middle-SDI regions. In contrast, epidemiological changes remain a significant factor hindering the reduction of the disease burden.

### 3.7 Risk factors

**Figure 7:**
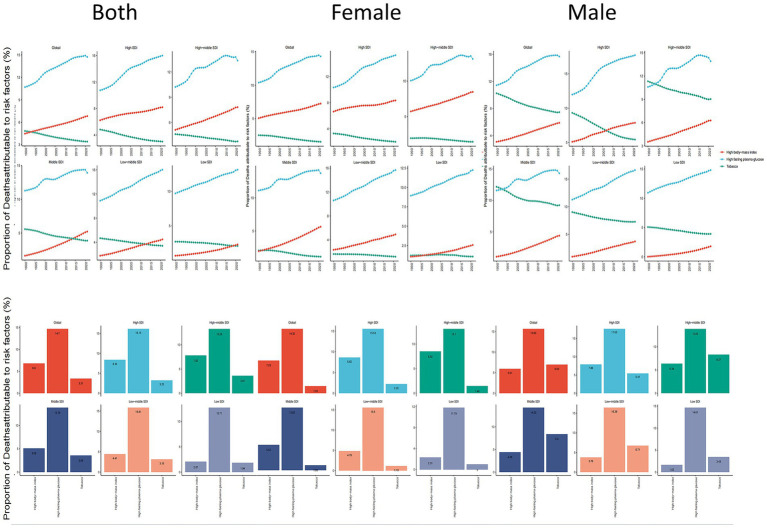
ADOD mortality risk factors by sex in global & 5 SDI regions, 1990–2021. ADOD, Alzheimer’s disease and other dementias.

Based on GBD 2021, high fasting blood glucose, high BMI, and tobacco use were identified as the leading risk factors for dementia. Globally, high fasting blood glucose contributed the highest proportion of mortality attributable to risk factors, at 14.70% (95% UI, 1.21–29.41), a trend that persisted across different SDI regions and sexes. In high-SDI regions, its impact was even greater, accounting for 16.15% (95% UI, 1.33–31.95) of ADOD mortality. Tobacco use ranked third globally and in all SDI regions (Figure 7).

Differences were observed in risk factors between genders and age groups. Smoking was the predominant risk factor for males, particularly in the 60–64 age group, where it accounted for 7.59% of mortality, although its impact decreased with age (Supplementary Figure 2). Meanwhile, the contributions of high fasting blood glucose and high BMI to mortality increased across both sexes and all age groups from 1990 to 2021, with little variation between age groups (Supplementary Figure 2).

## 4 Discussion

### 4.1 Overall findings

From 1990 to 2021, the global incidence, prevalence, and mortality rates of ADOD increased significantly by 156.54, 160.81, and 194.39%, respectively. Dementia is strongly age-associated, and its rising global incidence parallels increasing life expectancy (Li et al., 2022; Fu et al., 2024). Among the various subtypes, AD remains the most prevalent, accounting for 60–80% of all dementia cases worldwide (Tao et al., 2024). Furthermore, the World Health Organization (WHO) has identified dementia as the seventh leading cause of death worldwide (Fu et al., 2024; Alzheimer’s Disease Facts and Figures, 2024), underscoring its substantial public health impact. By 2,100, projections indicate that older adults will comprise over 60% of the population in 155 countries, with two-thirds of these individuals (approximately 1.1 billion) living in less developed regions (Jack et al., 2009). As populations continue to age, the burden of ADOD is intensifying, posing urgent challenges for healthcare systems, caregivers, and policymakers.

Our study confirms that the total number of ADOD cases, deaths, and DALYs has grown in nearly all regions. This increase, though varying in pace, can be attributed to multiple factors like a growing and aging population and evolving lifestyle habits. Notably, high-SDI regions continue to bear a disproportionately large share of the disease burden, likely due to a combination of extended life expectancy, more comprehensive diagnostic efforts, and lifestyle factors that increase ADOD risk (GBD 2016 Dementia Collaborators, 2019; GBD 2021 Osteoarthritis Collaborators, 2023).

### 4.2 Variations across SDI regions

ADOD burden trends diverged across SDI regions. High-middle-SDI regions exhibited the highest incidence (ASIR) and prevalence (ASPR), whereas low-SDI regions showed rapid increases in mortality (ASMR) and DALYs (ASDR). Countries such as China, Germany, and Lebanon recorded the highest ASIR and ASPR. China in particular experienced a notable rise in prevalence—from 17.37 million in 2019 to 19.12 million in 2021. Based on BAPC model projections, China’s ADOD prevalence could escalate by nearly 25%, reaching 24.09 million by 2050. With 14.9% of its population over 65 years old in 2022, the rapidly aging demographic in China underscores the need for comprehensive public health strategies and resources to mitigate ADOD’s impact (Chen et al., 2024; Liu et al., 2024).

### 4.3 Health inequality analysis

Significant absolute and relative inequalities in ADOD burden persist across SDI levels. While the slope index of inequality (SII) suggests that absolute inequalities have narrowed, the concentration index remained largely unchanged, implying stable relative disparities. In effect, high-SDI regions continue to shoulder a disproportionate share of the overall ADOD burden despite any improvement among lower-SDI countries. This pattern may be attributable to a combination of more advanced diagnostic infrastructure, demographic shifts favoring older populations, and lifestyle factors. Mitigating these persistent inequalities calls for targeted efforts: lower-SDI regions need further support to strengthen baseline healthcare capacity, while higher-SDI regions must refine dementia care pathways and resource use (Zare et al., 2015; Agudelo-Botero et al., 2023; Mouteyica and Ngepah, 2025).

### 4.4 Frontier analysis

Our frontier analysis, based on ASDR and SDI, underscores the potential for reducing ADOD burden in countries at various development levels. Notably, both low-SDI countries (e.g., Sierra Leone, Chad, Niger) and high-SDI countries (e.g., Belgium, Germany, Republic of Korea, the United States) exhibit substantial “improvement potential.” This indicates that economic development alone does not guarantee optimal outcomes and that targeted policy reforms are essential. For low-SDI countries, the gap may stem from inadequate healthcare coverage, insufficient diagnostic tools, and scarce financial resources (Dai et al., 2024). Policy efforts in these regions should therefore focus on strengthening primary care infrastructure, expanding diagnostic capacity through community-level programs, and leveraging international aid to increase healthcare funding. In high-SDI settings, the discrepancy between current performance and the theoretical frontier may reflect the need for more effective dementia management, broader adoption of preventive strategies, and better care models for aging populations. Overall, frontier analysis highlights that each nation, regardless of SDI category, can enhance ADOD outcomes by refining healthcare policies and maximizing resource utilization. This calls for policy changes aimed at integrating care systems, promoting early screening programs, and optimizing resource allocation to support preventative health and long-term care.

A critical consideration for our study is the limited inclusion of risk factors. While we identified high fasting blood glucose, high BMI, and tobacco use as leading contributors, these represent only a subset of the established risk factors for dementia. The GBD 2021 study identifies 12 established risk factors for dementia, but our analysis did not include others such as air pollution, less education, alcohol use, or traumatic brain injury. This limitation means that our findings on attributable burden are likely an underestimation of the total impact of risk factors on ADOD. Therefore, our results should be interpreted with this context in mind, and future research should aim to incorporate a more comprehensive range of risk factors to fully capture the complexity of the ADOD burden.

### 4.5 Decomposition analysis

Decomposition analysis underscores the paramount influence of aging and population growth on ADOD trends, jointly accounting for a significant share of DALY increases from 1990 to 2021. Aging alone was responsible for 58.13% of the global rise in DALYs, especially pronounced in middle-SDI regions. Additionally, epidemiological changes—including both preventive measures and the evolving natural history of dementia—play a mixed role. In high-SDI areas, these changes contributed to a relative decrease in ADOD burden (e.g., −1.75% for males and −2.24% for females), reflecting more robust healthcare systems and earlier interventions. By contrast, in lower-SDI regions, epidemiological transitions have not been sufficient to offset the strain from aging and population growth. Strengthening geriatric care, enhancing early detection, and reinforcing chronic disease management will remain critical strategies worldwide (Livingston et al., 2017).

### 4.6 Key risk factors and their implications

Pathological changes in dementia can begin 20 years or more before clinical symptoms emerge (Bateman et al., 2012; Quiroz et al., 2020), reinforcing the importance of addressing modifiable risk factors (Chen et al., 2024). According to GBD 2021, high fasting blood glucose, high BMI, and tobacco use are principal contributors to ADOD mortality. High fasting blood glucose accounts for the highest proportion of ADOD mortality attributable to risk factors—an impact modulated by both the age and duration of hyperglycemia (Pignalosa et al., 2021). Early and effective management of diabetes is therefore critical for minimizing cognitive decline and dementia progression. High BMI has consistently risen as a dementia risk factor in both males and females. Poor diet and obesity link to cognitive impairment throughout adulthood and in older age, possibly mediated by the gut microbiome, inflammatory pathways, and blood–brain barrier integrity (Jacobson et al., 2021; Pignalosa et al., 2021). Maternal obesity can also affect offspring’s cognitive development, though diets high in fiber may help mitigate these neurodevelopmental deficits by way of the gut-brain axis (Proctor et al., 2017; Liu et al., 2021). Tobacco use, while declining among males, remains paradoxical: nicotine can confer short-term cognitive benefits, but poses substantial risks, particularly in prenatal and adolescent populations (Leigh and Morris, 2020; Iiyama et al., 2023; Qiu et al., 2023). These findings emphasize the complexity of modifiable risk factors and the need for nuanced public health strategies focusing on early-life and midlife interventions.

### 4.7 Strengths of the study

In this study, we used the latest GBD database, which offers broader coverage and a longer time span than earlier versions, thereby enhancing the generalizability of our findings. We also combined frontier analysis, inequality indices, and decomposition analysis—an approach not previously applied to ADOD research—to identify key drivers and potential areas for improvement. These results not only deepen our understanding of how aging and cognitive decline intersect, but also provide policymakers with evidence-based guidance for resource allocation and targeted interventions. For example, our decomposition analysis indicates that focusing on aging management and glycemic control in middle-SDI regions could significantly curb the escalating ADOD burden.

### 4.8 Limitations

The present study has several limitations. First, our findings are subject to the methodological constraints of the GBD 2021 study (GBD 2021 Stroke Risk Factor Collaborators, 2024). While various techniques were employed to reduce bias and enhance accuracy, the potential for bias cannot be entirely eliminated. Second, although 12 risk factors associated with dementia have been identified (Livingston et al., 2020), GBD 2021 only includes three (high fasting blood glucose, high BMI, and tobacco use). This limited scope may lead to an underestimation of the total attributable burden of ADOD, as other significant factors such as less education, hearing loss, air pollution, and traumatic brain injury were not accounted for. Consequently, our findings on risk factor contribution, while significant, are not comprehensive. Third, the GBD 2021 dataset does not differentiate between dementia subtypes, such as vascular dementia or dementia with Lewy bodies, which reduces the robustness and clinical relevance of the findings. Given that different dementia subtypes vary in disease burden and associated risk factors, the absence of subtype data represents a significant limitation. Additionally, diagnostic criteria, biomarkers, medical records, and insurance codes for dementia have evolved over the past three decades, potentially introducing heterogeneity in the data. As a result, discrepancies between our analysis and real-world data are inevitable.

## 5 Conclusion

In summary, ADOD poses a critical, rapidly evolving challenge to public health worldwide, driven largely by aging populations and compounding risk factors such as diabetes, obesity, and tobacco use. The analyses presented—encompassing incidence and mortality trends, frontier evaluations, inequality assessments, and risk factor decomposition—collectively underscore the need for multifaceted, context-specific strategies to mitigate the ADOD burden. Strengthening healthcare infrastructure, prioritizing prevention and early intervention, and fostering international collaboration remain key to addressing existing disparities and achieving more equitable outcomes for aging populations globally.

## Data Availability

The original contributions presented in the study are included in the article/Supplementary material, further inquiries can be directed to the corresponding author.

## Footnotes

^1^ http://ghdx.healthdata.org/gbd-results-tool

^2^ https://www.who.int/data/gho/indicator-metadata-registry/imr-details/2380
