# Supplementary material for: Global burden of Alzheimer’s disease and other dementias (1990–2021): inequality, frontier, and decomposition analysis
Source: Front Aging Neurosci. 2025 Sep 18;17:1637029. doi: 10.3389/fnagi.2025.1637029 (PMC12489941; doi:10.3389/fnagi.2025.1637029)
Supplement: Supplementary file 1 [file Supplementary_file_1.docx]

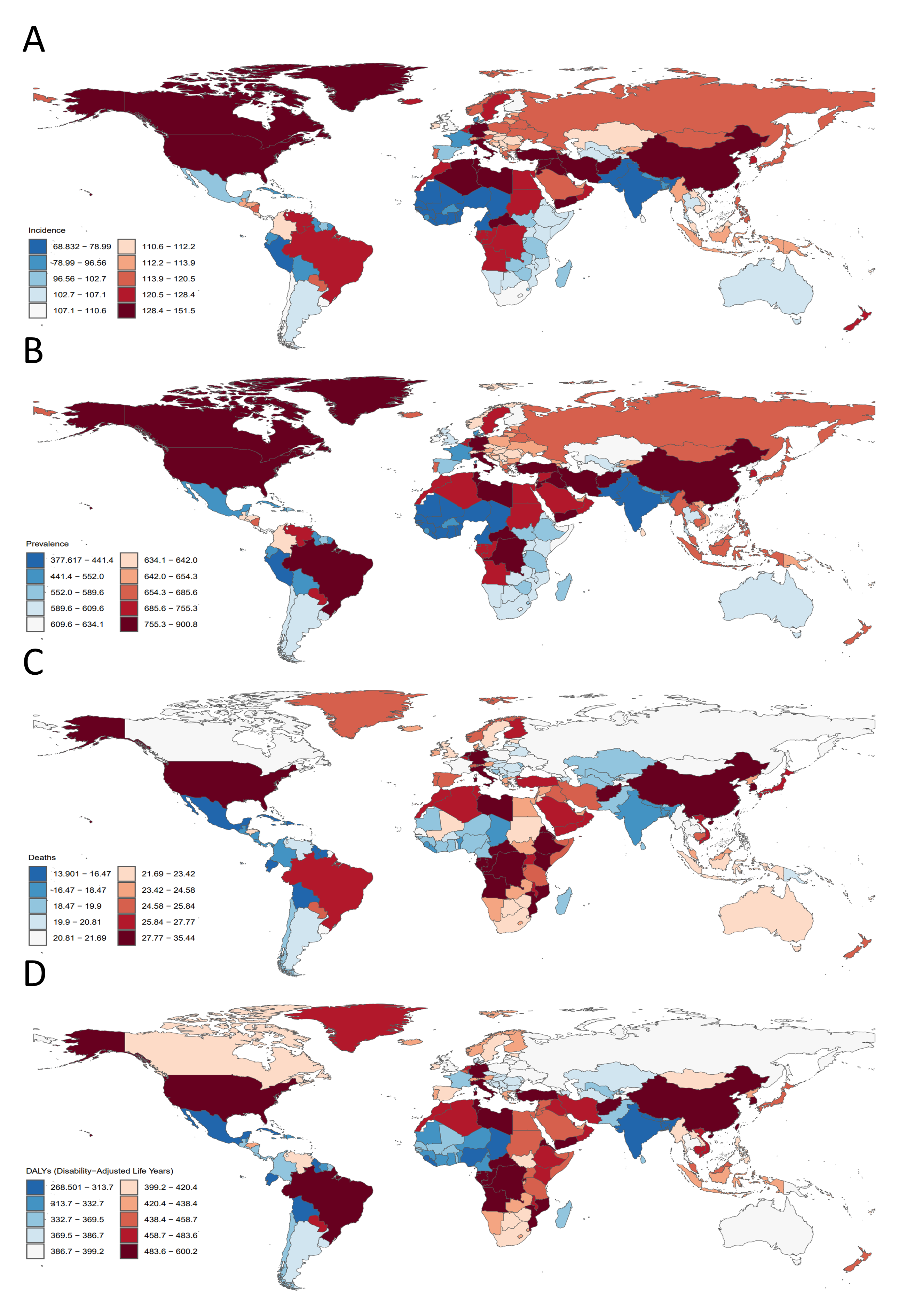


Supplementary Figure 1 EAPC in ASIR, ASPR, ASMR, and ASDR of ADOD Across 204 Countries.

Abbreviation: ADOD, Alzheimer's disease and other dementias; ASDR, age-standardized disability-adjusted life years rate; ASIR, age-standardized incidence rate; ASMR, age-standardized mortality rate; ASPR, age-standardized prevalence rate; DALY, disability-adjusted life-years; EAPC, estimated annual percentage change.


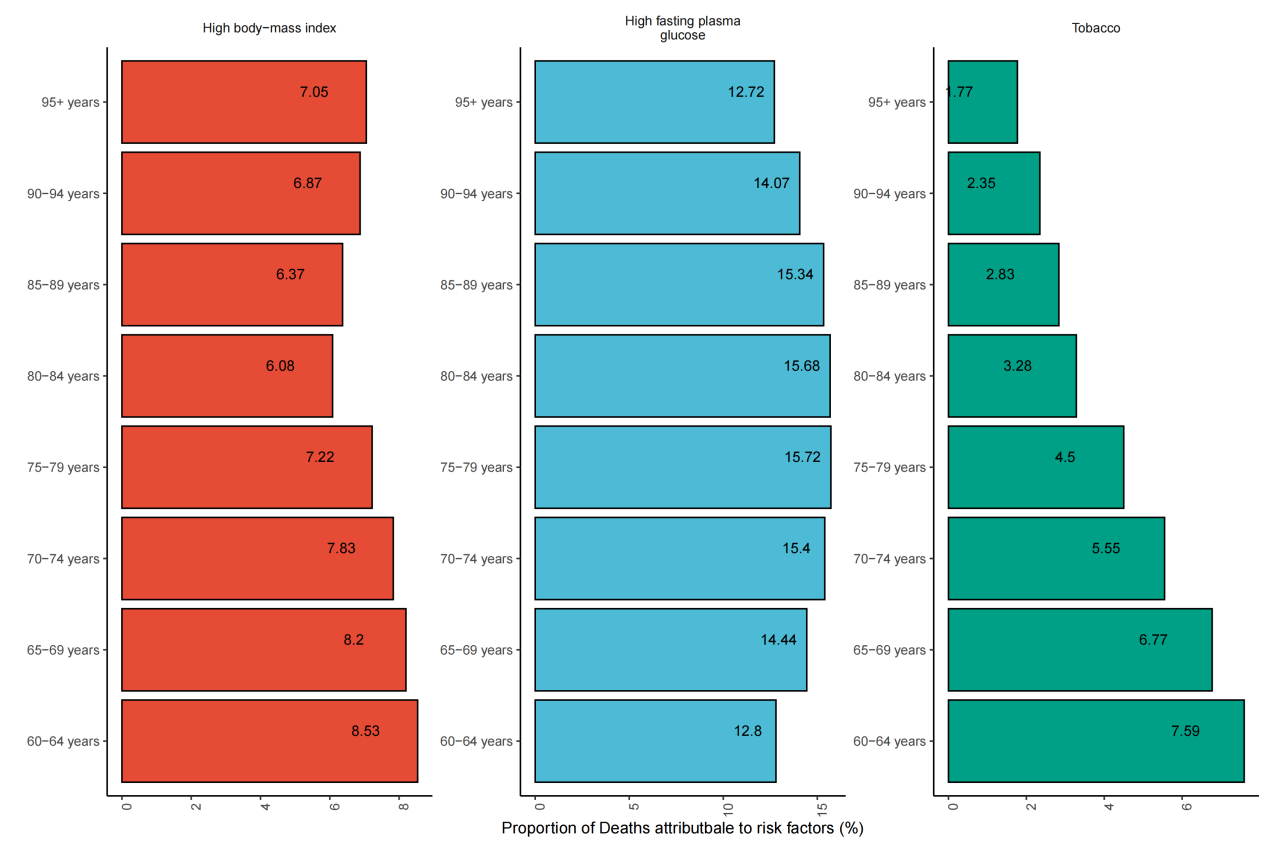


Supplementary Figure 2 ADOD Mortality Risk Factors by age globally in 2021.

Abbreviation: ADOD, Alzheimer's disease and other dementias.
